# Sequential evaluation of a robotic dry-bone resection system using virtual model control with integral control: an exploratory benchtop study

**DOI:** 10.1007/s11701-026-03963-9

**Published:** 2026-09-22

**Authors:** Ryan McWilliams, Daniel Larby, Fulvio Forni, Vikas Khanduja

**Affiliations:** 1 Department of Surgery, University of Cambridge, Cambridge, UK; 2 Department of Surgery, Addenbrooke’s Hospital, Cambridge University Hospitals NHS Foundation Trust, Cambridge, UK; 3 Department of Engineering, University of Cambridge, Cambridge, UK

**Keywords:** Robotic surgery, Orthopaedic robotics, Bone resection, Virtual model control, Integral control, Trajectory accuracy

## Abstract

Virtual model control (VMC) provides compliant trajectory execution, but sustained contact loads can produce residual positional error. This study evaluated the sequential development of VMC augmented with integral control for powered dry-bone resection and assessed the effects of end-effector stabilisation, spatial calibration and operating parameters. A seven-degree-of-freedom Franka Research 3 robot was commanded using VMC with integral control and carried a 4-mm diamond-tipped arthroscopic burr through a programmed 10 × 10 mm serpentine trajectory in 20 pounds-per-cubic-foot synthetic bone. Four sequential configurations were assessed: an original handle-based mount, an anthropomorphically designed shaft-stabilising mount, robot-frame calibration and workpiece registration. Each configuration comprised three resections. Performance was graded by one non-blinded assessor using a study-specific twelve-point score across burr-tip oscillation and X-, Y- and Z-axis deviation, with lower scores indicating better performance. Radial depth of cut, engagement depth, trajectory velocity and burr angle were then screened to identify candidate operating parameters. Mean total score decreased sequentially from 11.7/12 with the original mount to 7.7/12 after shaft stabilisation, 5.7/12 after robot-frame calibration and 4.0/12 after workpiece registration. The lowest observed score was maintained at an RDOC between 0.10 and 0.25 mm, a measured depth between 1.52 and 1.89 mm, velocity between 1.5 and 2.5 mm/s and a burr angle between 30 and 45 degrees. Mechanical stabilisation and spatial calibration were associated with progressively better resection performance using VMC with integral control. This exploratory benchtop study identified candidate operating ranges for further assessment in higher-fidelity models.

## Introduction

Cam-type femoroacetabular impingement (FAI) syndrome is a recognised cause of hip pain and impaired function in young and active adults [1]. International consensus defines FAI syndrome by the triad of appropriate symptoms, clinical signs and imaging findings [2]. It is associated with loss of sphericity at the femoral head–neck junction, which can produce abnormal contact with the acetabular rim and contribute to labral and chondral injury [3, 4]. Prospective cohort evidence has also associated cam morphology with the development and progression of hip osteoarthritis [5]. Arthroscopic femoral osteochondroplasty aims to restore the physiological head–neck contours and normalise joint contact stresses [6–8]. Randomised controlled trials have reported superior short-term patient-reported outcomes following arthroscopic surgery compared with structured non-operative treatment [9, 10], while registry data have demonstrated significant early improvement in patient-reported outcomes [11]. However, achieving an accurate resection remains technically challenging [12]. The procedure has a recognised learning curve [13, 14], as arthroscopy provides only a restricted view of the femoral head–neck junction at any one time, requiring the surgeon to translate a three-dimensional correction into burr movements through a constrained portal. Insufficient bone removal may leave residual cam morphology, which is the leading indication for revision hip arthroscopy [15–17]. Revision rates of approximately 5% have been reported, with incomplete bony resection implicated in up to 80% of revision procedures [16, 17]. Conversely, excessive resection can reduce femoral-neck strength and increase the risk of fracture [18–20] and has been associated with inferior outcomes after revision surgery [21]. These risks of under-resection and over-resection create a need for technologies that can both define and execute bone removal more consistently.

Computer-assisted navigation and robotic systems aim to translate a predefined surgical plan into more precise intraoperative guidance or movement. Navigation supports intraoperative spatial guidance, whereas robotic systems can actively constrain or execute tool motion [22]. Previous investigations in synthetic-bone models and clinical cohorts have reported improved accuracy of cam correction using these approaches [23–27]. Navigation guides rather than controls the resection: the surgeon remains responsible for manipulating the burr, leaving the accuracy of plan execution susceptible to human error [12, 23]. Navigation therefore does not guarantee complete reproduction of the preoperative plan. In one study, under-resection and over-resection were still observed at 5.1% and 3.6% of sampled points, respectively, with maximum deviations of 7.1 mm and 6.3 mm from the planned model despite navigation assistance [27]. Consequently, robotics offers the potential to combine surgical planning with controlled execution of the resection trajectory. However, even when an appropriate trajectory has been defined, the accuracy of its execution depends on the dynamic interaction between the burr and bone, an interaction to which human surgeons can adapt intuitively but that a robotic system must accommodate through its mechanical and control architecture.

The robotic platform examined in this study used virtual model control (VMC) with integral control. Impedance control provides a general framework for regulating the dynamic relationship between motion and force during contact between a manipulator and its environment [28]. VMC implements compliant interaction by representing the intended Cartesian motion as a virtual mechanism connected to the physical end effector through simulated stiffness and damping [29, 30]. Differences in position and velocity between the virtual reference and the end effector generate corrective forces that are mapped to the robot’s joints. Although this compliant coupling facilitates surface interaction, a sustained contact force can deflect the end effector from its reference position. The integral control component accumulates this residual positional error and progressively increases the corrective response [31].

The present study evaluated the dynamic interaction between the burr and bone using VMC with integral control as the robotic control architecture. In contrast to conventional navigation, which primarily assists the surgeon in interpreting and manually executing a plan, the investigated platform used a programmed tool path to command robotic burr movement. The principal novelty of this work lies in applying VMC with integral control to execute powered robotic bone resection along a programmed tool path using an arthroscopic burr. The system was assessed through successive stages incorporating mechanical stabilisation of the burr shaft, robot-frame calibration and workpiece registration. The primary objective was to determine how these cumulative modifications were associated with burr oscillation and deviation from the programmed tool path. The secondary objective was to identify suitable ranges of radial depth of cut (RDOC), engagement depth, cutting velocity and burr angle for subsequent testing in an arthroscopic hip phantom.

## Materials and methods

### Study design

A staged benchtop study evaluated five sequential system conditions. Condition I assessed dry-bone resection using the original burr mount. Condition II incorporated a custom anthropomorphically designed shaft-stabilising mount. Condition III incorporated spatial calibration between the software model and the physical robot, while Condition IV added registration of the operating surface. Under Condition V, four operating parameters were assessed individually to identify candidate settings for subsequent investigation.

### Robotic platform and cutting system

**Fig. 1:**
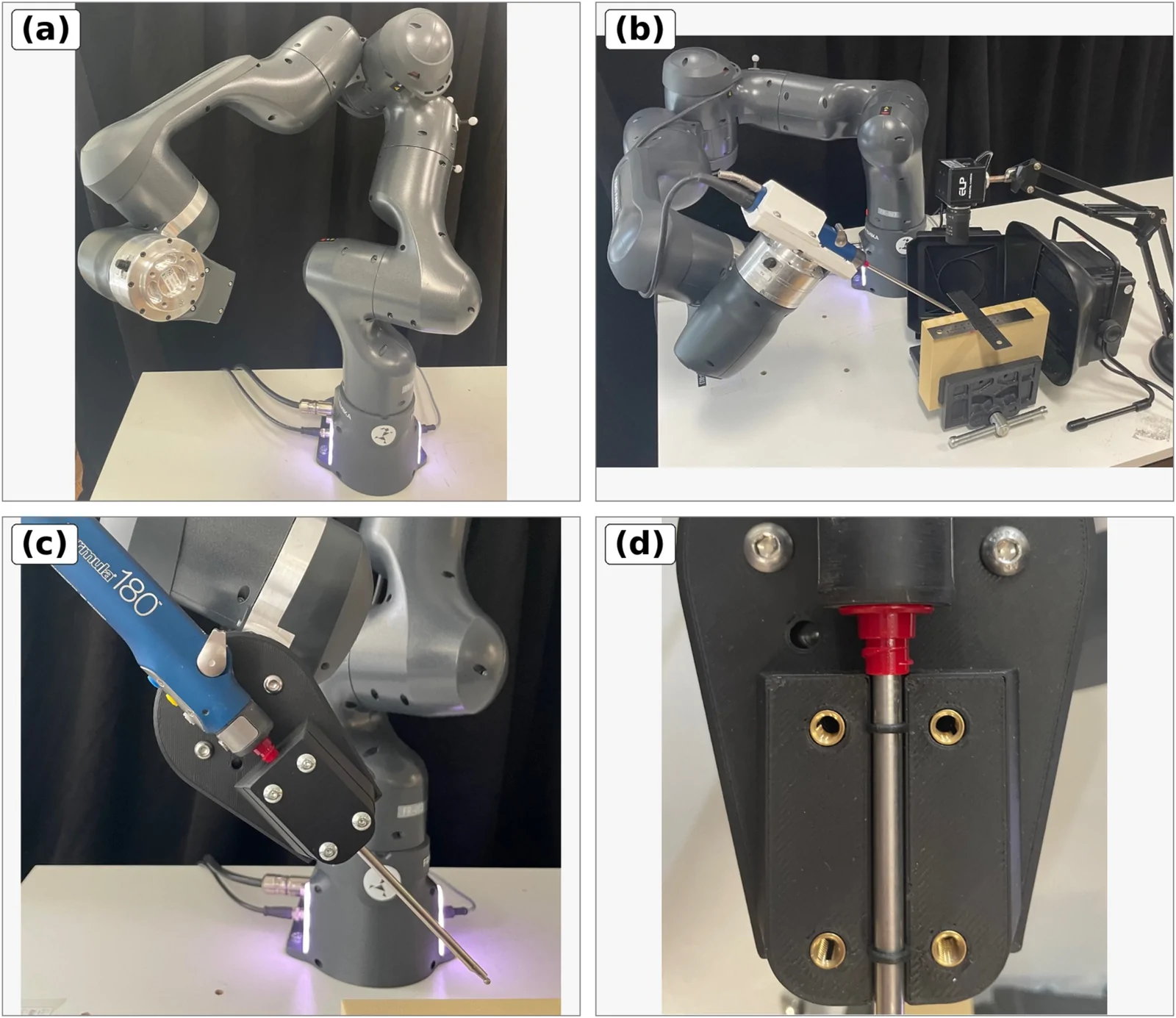
Experimental platform and development of the burr-mounting system. (**a**) Franka Research 3 robot used for benchtop testing. (**b**) Original handle-based burr-mounting configuration with a fixed synthetic-bone block. (**c**) Revised custom mount supporting the proximal burr shaft while leaving the distal shaft exposed for bone engagement. (**d**) Close-up of the mount interior, showing the proximal shaft support and the two O-rings surrounding the burr shaft

A seven-degree-of-freedom Franka Research 3 robotic arm (Franka Emika, Munich, Germany) was used. The robot carried a Stryker CrossBlade XL Diamond Burr (Stryker, Kalamazoo, USA). The 4-mm diamond burr was operated at 12,000 revolutions per minute. A synthetic bone block with a density of 20 pounds per cubic foot (PCF) was fixed within a benchtop vice. The complete benchtop arrangement is shown in Fig. 1a.

The original mount primarily constrained the burr handle. Because the burr incorporated a removable shaft, compliance was observed at the shaft-handle interface. A revised mount was designed using three-dimensional computer-aided design and manufactured by three-dimensional printing. Inspired by the grip used by hip-preservation surgeons, the mount enclosed and supported the proximal shaft while retaining sufficient exposed distal shaft for future arthroscopic access. Two rubber O-rings restricted rotation within the mount and reduced transmission of vibration. The original and revised mounting arrangements are shown in Fig. 1.

### Virtual model control with integral control and programmed trajectory

The motion trajectory and VMC with integral control were implemented in the Julia programming language using Visual Studio Code (Microsoft, Washington, USA). The desired trajectory was represented by a virtual Cartesian ‘cart’, and the physical end effector was coupled to this reference through virtual stiffness and damping.

The end effector moved from an idle pose to a predefined task-start pose while achieving the required orientation at a velocity proportional to the distance from the start pose. It then approached the bone along the Z-axis at 2.5 mm/s. The burr was activated manually before contact. A serpentine raster trajectory was executed in which the burr traversed the 10-mm square along the X-axis, advanced in the negative Y-direction by the programmed RDOC, returned along the X-axis and repeated the cycle until the square was covered. It then withdrew along the Z-axis before the burr was deactivated. The burr was maintained at 45 degrees to the bone surface in Experiments I-IV.

### Sequential mechanical modification and spatial calibration

Four sequential system configurations were evaluated, with three 10 × 10 mm square resections performed for each configuration. The initial condition (Experiment I) used a burr mount attached to the handle of the burr, whereas the second condition (Experiment II) incorporated a custom shaft-stabilising mount. Experiment III included spatial calibration of the robot using infrared reflective markers attached to the robot links and end effector. An OptiTrack motion-capture system (NaturalPoint Inc., Corvallis, USA) measured the positions and orientations of the marker configurations, enabling alignment of the robot-base, end-effector and virtual-model coordinate frames. In the final condition (Experiment IV), the workpiece was registered by positioning the burr tip at four distinct points distributed across three surfaces of the synthetic-bone block. These measurements defined the position and orientation of the operating zone, thereby enabling alignment of the programmed resection trajectory with the physical bone surface and reducing positional error associated with manual placement of the block within the benchtop vice.

### Operating-parameter characterisation

Following mechanical stabilisation and spatial calibration, Experiment V assessed four parameters individually while the remaining parameters were held at their standard settings: RDOC, programmed engagement depth, trajectory velocity and the angle between the burr shaft and bone surface. The parameters were tested in the following order, with a single resection performed at each setting because the intended resection depths could not be reproduced with sufficient accuracy to permit reliable replication. RDOC values of 0.10, 0.15, 0.20, 0.25, 0.30 and 0.40 mm were tested. The attempted resection depths were 0.50, 1.00, 1.50, 2.00, 2.50 and 3.00 mm, and the corresponding measured depths were 0.64, 0.86, 1.52, 1.89, 2.67 and 3.06 mm, respectively. Depth was measured using a depth gauge. Velocities of 1.5, 2.0, 2.5, 3.0 and 3.5 mm/s and burr angles of 15, 30, 45, 60 and 75 degrees were assessed.

### Outcome assessment

Tool-path deviation was defined as the linear difference between the programmed and observed burr-tip position arising during robot-bone interaction. Oscillation was defined as visible vibration of the burr tip during contact. A single non-blinded assessor reviewed slow-motion footage frame by frame. Rulers aligned with the X- and Y-axes provided a scale, and the camera was positioned above the operating zone to reduce perspective distortion. Z-axis deviation was assessed using measured resection depth. The resected surfaces and video footage were reviewed for endpoint deviation, and the difference between physical and programmed endpoints was recorded when deviation occurred in the direction of travel.

Four domains were graded: visible oscillation, X-axis deviation, Y-axis deviation and Z-axis deviation. Each received 0 points for no measurable oscillation or deviation, 1 point for approximately 1 mm, 2 points for approximately 2 mm and 3 points for 3 mm or greater. Domain scores were summed to produce a total from 0 to 12, with lower scores indicating closer adherence to the planned trajectory. The total score provided a single descriptive value that could be easily compared between experiments. Because the high-frequency burr-tip movement exceeded the temporal resolution needed for plane-specific vibration analysis, oscillation was graded as a single overall domain rather than separately in each axis.

### Analysis

Results were summarised descriptively. For Experiments I-IV, individual total scores and means are reported. Formal hypothesis tests and confidence intervals were not used because each sequential configuration comprised three resections, the composite outcome was ordinal and the study was not designed or powered for inferential comparisons. Experiment V observations were used to define candidate settings for subsequent replicated testing.

## Results

### Initial powered resection

All three squares in Experiment I demonstrated visible oscillation and deviations in each spatial axis. X- and Y-axis deviations were at least 3 mm in every attempt; Z-axis deviation was at least 3 mm in two attempts and 2 mm in one. Total scores were 12, 11 and 12 (mean 11.7).

### Effect of shaft stabilisation and calibration

**Fig. 2:**
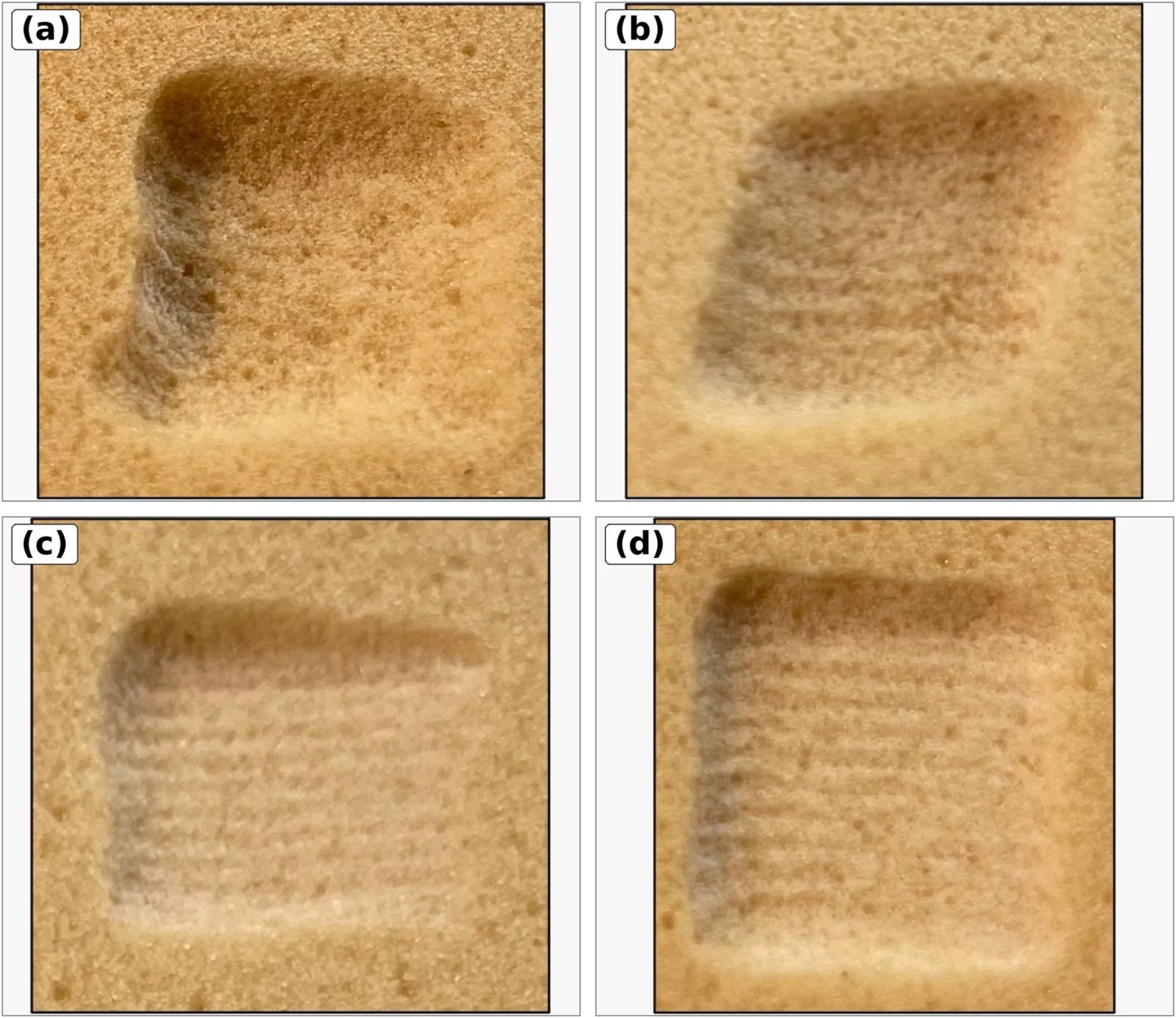
Progressive changes in dry-bone resection during sequential system development. Panels (**a**)-(**d**) show representative resections from Experiments I-IV, respectively. The sequence illustrates the transition from an irregular resection to a more clearly defined square

In Experiment II, the shaft-stabilising mount reduced visible burr-tip oscillation to approximately 1 mm in all three resections. X- and Y-axis deviations were 2 mm, and Z-axis deviation remained between 2 and 3 mm. Total scores were 8, 7 and 8 (mean 7.7). The resection surface changed from a cratered topography to a smoother, wave-like finish, although the intended square exhibited a rhomboid appearance (Fig. 2a and b).

**Fig. 3:**
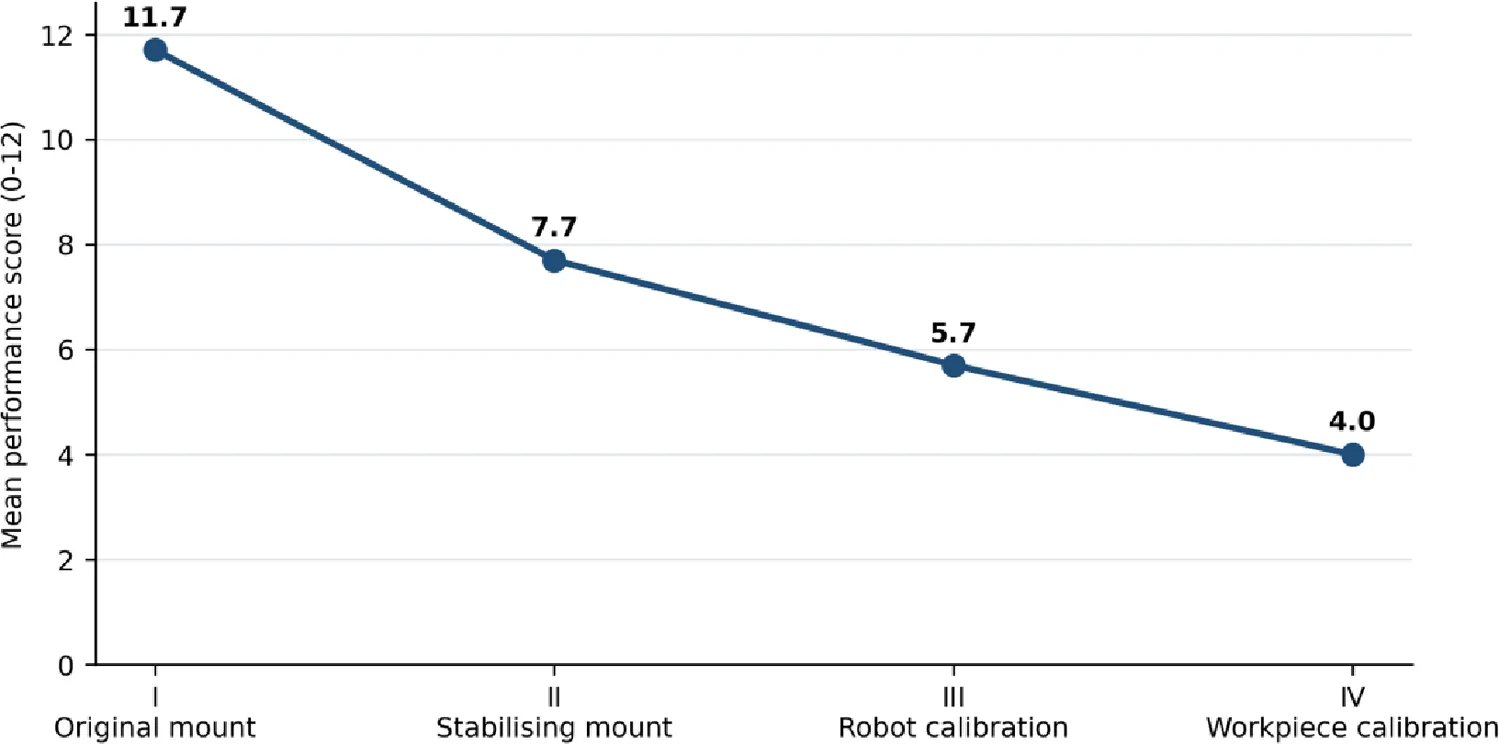
Sequential improvement in mean performance score. Each configuration comprised three 10 × 10 mm resections. Lower scores indicate less oscillation and trajectory deviation

In Experiment III, robot-frame calibration produced further improvement, with mean X- and Y-axis scores of 1.3 and a mean Z-axis score of 2.0. Total scores were 6, 5 and 6 (mean 5.7). Surface topography became smoother, although one side remained deeper and produced a scalloped base. After workpiece registration in Experiment IV, every resection scored 1 in all four domains, giving a mean score of 4. The resulting squares had a visually level base, more linear margins and corners that more closely approximated right angles. These sequential changes in surface appearance are shown in Fig. 2. The mean total score decreased by 7.7 points between Experiments I and IV (Table 1; Fig. 3).

**Table 1:** Domain and total performance scores across sequential development

Experiment	Configuration	Oscillation	X	Y	Z	Total score
I	Original mount	3.0	3.0	3.0	2.7	11.7 (12, 11, 12)
II	Stabilising mount	1.0	2.0	2.0	2.7	7.7 (8, 7, 8)
III	Robot calibration	1.0	1.3	1.3	2.0	5.7 (6, 5, 6)
IV	Workpiece registration	1.0	1.0	1.0	1.0	4.0 (4, 4, 4)

Key: Domain and total values are means; individual total scores are shown in parentheses. Lower scores indicate better performance

### Operating-parameter characterisation

In Experiment V, the lowest observed total score of 4 was achieved across RDOC values of 0.10–0.25 mm. Scores increased to 5 at 0.30 mm and 6 at 0.40 mm, driven mainly by Y- and Z-axis deviations. Although RDOC values as low as 0.10 mm maintained the same observed score, 0.20–0.25 mm provided the preferred balance between efficiency and performance.

For depth, the best score of 4 occurred at measured depths of 1.52 and 1.89 mm. Shallower measured depths of 0.64 and 0.86 mm scored 5 because oscillation increased to 2 mm. Deeper cuts of 2.67 and 3.06 mm scored 6, with increased Y- and Z-axis deviation. The mean absolute error between attempted and measured depth was 0.11 mm.

**Fig. 4:**
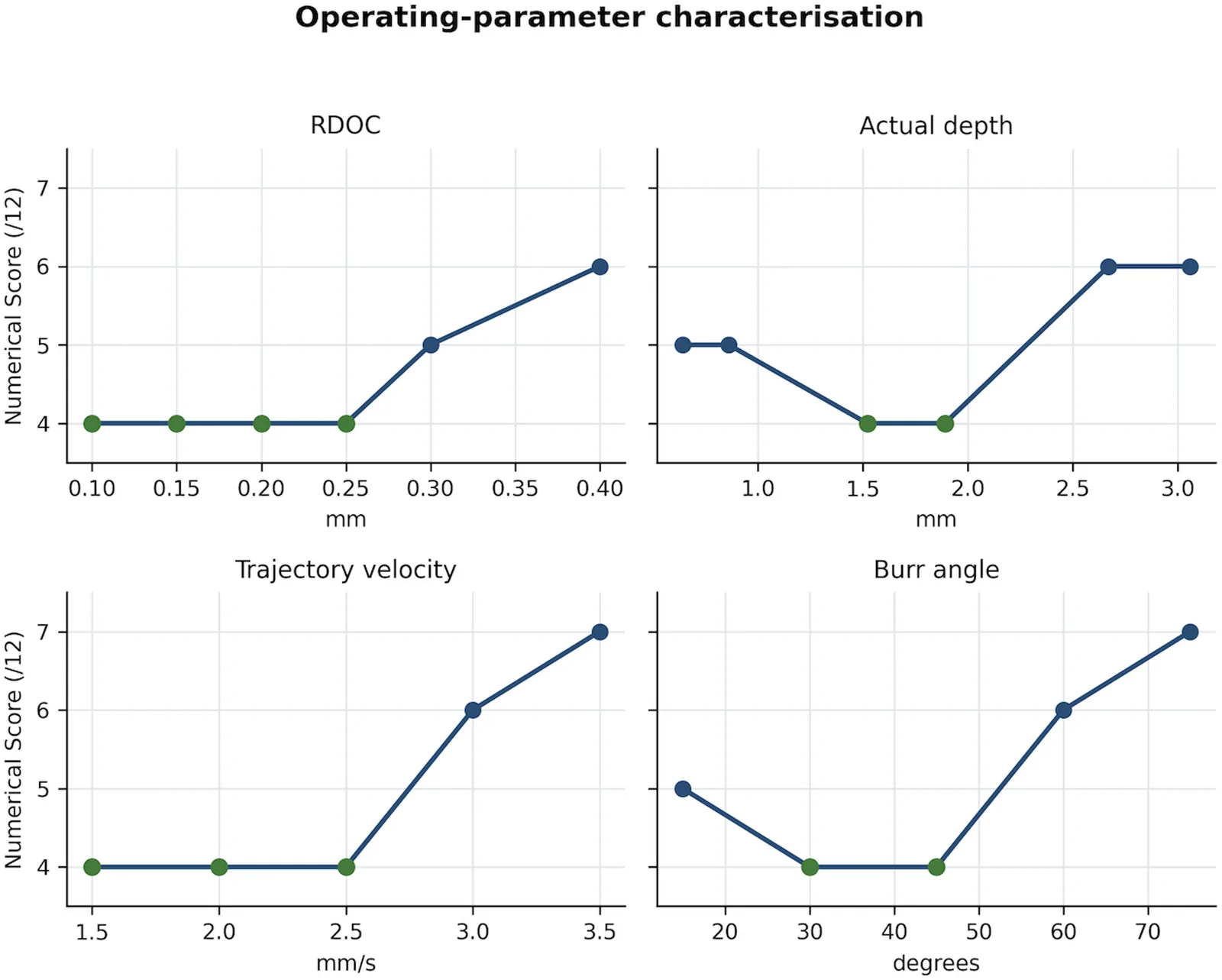
Performance score across RDOC, measured engagement depth, trajectory velocity and burr angle. Green markers indicate settings attaining the best observed score of 4. Each parameter was varied individually. The composite score ranged from 0 to 12, with lower scores indicating closer adherence to the planned trajectory

Velocities of 1.5, 2.0 and 2.5 mm/s each scored 4. Performance deteriorated at 3.0 mm/s (score 6) and 3.5 mm/s (score 7), with increased oscillation and spatial deviation. Burr angles of 30 and 45 degrees scored 4; performance was lower at 15 degrees (score 5), 60 degrees (score 6) and 75 degrees (score 7). The recorded data therefore supported candidate operating ranges of RDOC 0.10–0.25 mm, measured depth 1.52–1.89 mm, velocity 1.5–2.5 mm/s and angle 30–45 degrees. Efficiency considerations favoured RDOC 0.20–0.25 mm and velocity 2.0–2.5 mm/s (Fig. 4).

## Discussion

This study describes the sequential development and exploratory evaluation of VMC with integral control for dry-bone resection. First, the original handle-based mount did not adequately constrain the burr shaft during bone contact; supporting the proximal shaft was associated with less visible oscillation and a lower performance score. Second, robot-frame calibration followed by workpiece registration produced further incremental changes, particularly in Z-axis depth uniformity. Third, the parameter screen identified candidate ranges in which the lowest observed score was maintained: RDOC 0.10–0.25 mm, measured depth 1.52–1.89 mm, velocity 1.5–2.5 mm/s and angle 30–45 degrees. Across the sequential configurations, the mean score decreased from 11.7 to 4.0.

Contact between the diamond-tipped burr and synthetic bone produced rapid, irregular displacement of the burr tip. We used the descriptive term ‘stochastic jumping’ for this behaviour, hypothesising that contact depended on the distribution of bone particles, the orientation at which individual diamond particles contacted the bone, and the position and orientation of each particle within the resection zone. When the shaft-handle interface remained compliant, motion was not transmitted rigidly to the burr tip. The tip lagged behind the mount, particularly at direction changes, contributing to acute cornering and unresected bone islands.

The shaft-stabilising mount was deliberately surgeon-inspired. Experienced surgeons were observed to stabilise the burr closer to the shaft rather than relying solely on the handle, shortening the unsupported shaft. The revised mount reproduced this principle and incorporated O-rings to resist rotation and damp vibration. Adding mechanical support improved the average performance score by four points and produced a smoother resection surface.

Robot-frame calibration aligned the software representation with the physical base and end-effector frames. X- and Y-axis scores were lower after this stage, but the resection remained deeper on one side because the manually positioned workpiece was not sufficiently aligned with the robot’s physical base and end-effector frames. Registration of three workpiece planes then allowed the trajectory to be expressed relative to the operating zone, and the mean Z-axis score decreased from 2.0 to 1.0. This distinction between robot calibration and workpiece registration is consistent with the literature, in which alignment among image, tracker, instrument and patient coordinate frames is central to executing a planned resection [27, 32].

Increasing RDOC increased the resected volume during each pass and was associated with greater lateral and depth deviation above 0.25 mm. Engagement beyond approximately half the 4-mm burr diameter created an overhanging bone edge that could be struck during oscillation, which may explain the higher scores at measured depths above 2 mm. Conversely, very shallow cuts showed more oscillation and would require additional passes. Velocity above 2.5 mm/s may have reduced the time available for the compliant system to establish stable contact with the bone and was associated with worse performance scores. Angles between 30 and 45 degrees provided the lowest scores within the tested range; steeper angles increased lateral and depth deviation, while the shallowest angle increased X-axis deviation. However, as each parameter was varied independently, the data do not establish interactions among the parameters; therefore, only candidate reference values can be inferred.

This work identified two requirements for future iterations. First, calibration accuracy declined during repeated testing and required periodic manual recalibration. An optical tracking system is recommended to remain active during resection and continuously measure the positional error between the burr tip and the workpiece. Secondly, this optical tracking system should be programmed to provide a more objective outcome measure and allow tracking error to be analysed over time and by axis, as millimetre-level slow-motion footage grading is insufficient for detailed controller optimisation.

This study has several limitations. Each sequential configuration was assessed using only three resections, the performance-based scoring system was an unvalidated outcome measure tool, and assessment was performed by a single non-blinded assessor. Consequently, the reliability of these findings is limited, and repeatability could not be robustly assessed. Formal statistical inference was therefore not appropriate, and the observed changes should be interpreted as descriptive findings rather than proof of superiority. Future studies should use motion-tracking error and surface-topography analyses to improve the objective assessment and reporting of resection performance, which could be achieved through the use of optical trackers. Additionally, because the modifications were introduced cumulatively, the observed differences between configurations may have been influenced by experimental order, burr wear and calibration drift. The study therefore evaluates the performance of the progressively modified system but does not isolate the independent contribution of any single modification. Finally, the homogeneous 20-PCF block did not reproduce anatomy, cortical–cancellous transitions, soft tissue or an arthroscopic portal.

## Conclusion

The findings support the feasibility of executing a programmed bone-resection trajectory using a robotically controlled arthroscopic burr. Sequential mechanical stabilisation, spatial calibration and workpiece registration enabled a robotic arm commanded by VMC with integral control to progress from an unstable resection to a more precisely resected square with an improved resection performance score in a synthetic-bone block. The mean performance score decreased from 11.7/12 to 4.0/12 after the cumulative modifications. The lowest observed score was maintained at an RDOC of 0.10–0.25 mm, a measured engagement depth of 1.52–1.89 mm, a trajectory velocity of 1.5–2.5 mm/s and a burr angle of 30–45 degrees. Efficiency considerations favoured an RDOC of 0.20–0.25 mm and a velocity of 2.0–2.5 mm/s. These ranges will be considered as candidate operating settings for future resections in arthroscopic phantoms. However, because of the small number of resections performed, the reliability of these findings is limited, and repeatability could not be robustly assessed. Future work should incorporate continuous optical tracking and quantitative assessments of resection-volume error, surface topography, completion time and burr-tip tracking error. Replicated testing in higher-fidelity hip phantoms and cadaveric specimens under arthroscopic access is required before the system’s safety, reproducibility and clinical relevance can be established. Such studies should determine whether the improvements observed in this benchtop model translate into clinically meaningful gains in resection accuracy and consistency.

## Data Availability

No datasets were generated or analysed during the current study.
